# Gas Damping in Capacitive MEMS Transducers in the Free Molecular Flow Regime

**DOI:** 10.3390/s21072566

**Published:** 2021-04-06

**Authors:** Boris A. Boom, Alessandro Bertolini, Eric Hennes, Johannes F. J. van den Brand

**Affiliations:** 1 National Institute for Subatomic Physics, Science Park 105, 1098 XG Amsterdam, The Netherlands; alberto@nikhef.nl (A.B.); ehennes@nikhef.nl (E.H.); jo@nikhef.nl (J.F.J.v.d.B.); 2 Faculty of Science and Engineering, Maastricht University, Minderbroedersberg 4–6, 6211 LK Maastricht, The Netherlands

**Keywords:** capacitance transducers, free molecular flow, gas damping, Monte Carlo methods, Q measurement

## Abstract

We present a novel analysis of gas damping in capacitive MEMS transducers that is based on a simple analytical model, assisted by Monte-Carlo simulations performed in Molflow+ to obtain an estimate for the geometry dependent gas diffusion time. This combination provides results with minimal computational expense and through freely available software, as well as insight into how the gas damping depends on the transducer geometry in the molecular flow regime. The results can be used to predict damping for arbitrary gas mixtures. The analysis was verified by experimental results for both air and helium atmospheres and matches these data to within 15% over a wide range of pressures.

## 1. Introduction

Accurate prediction of the gas damping in microelectromechanical systems (MEMS) structures is of vital importance for the design of various types of microsensors. For instance, the accuracy and resolution of MEMS gyroscopes is closely linked to the quality factor of the vibrating sensing elements, and the Brownian noise level in MEMS accelerometers is often dominated by squeezed-film damping in the narrow gaps in its capacitive transducers. To obtain sufficiently high quality factors, these devices are usually encapsulated in a package at reduced pressures. Because of the typically small feature sizes in these microsensors, the mean free path of the gas molecules inside these packages is often significantly larger than the relevant dimensions of the sensor, and the gas damping is fully determined by individual gas–wall collisions.

Generally speaking, there are two classes of models for gas damping in this free molecular flow regime, both with their own challenges. On the one hand, there are integral equation models based on the collisionless Boltzmann equation. Closed-form solutions are hard to obtain for anything but the most simple of geometries [1]. Bao et al. [2] attempted to incorporate the increased damping caused nearby stationary walls, but these results underestimate this squeezed-film damping as observed in experiments. Several limiting assumptions were subsequently removed by Frangi et al. [3], resulting in a deterministic 3D model for damping in the rarefied gas regime assuming fully diffuse gas–wall interactions. An overview of this approach in context to related work can be found in [4].

On the other hand, there is an approach that uses Monte-Carlo methods to infer the behavior of the total system from the simulated trajectories of a large number of typical test particles. Efficient simulation methods for rarefied gases were developed by Bird [5]. Typical studies use a numerical implementation of the molecular dynamics to track the energy transfer of many individual molecules to moving bodies [6,7]. Both of these classes of approaches prove to give accurate results, but they require developing custom simulation tools and provide little insight into how the damping changes with geometry.

Here, we present an analysis that is based on relatively simple analytical models for both kinetic gas damping [8] and squeezed-film damping [9] in the free molecular flow regime. The latter is assisted by straightforward Monte-Carlo simulation of the 3D geometry under consideration in Molflow+, a freely available simulation tool which can include the effect of incomplete momentum and thermal accommodation. The resulting estimate for the total gas damping matches experimental results over a wide range of pressures and capacitive transducer gaps. These results provide insight into the dependence of squeezed-film damping on the geometry of capacitive transducers that can be helpful in designs where the trade-off between gas damping and electrical performance of the capacitive transducers is critical.

## 2. Materials and Methods

### 2.1. Damping Contributions

In a gas, the average distance travelled by individual molecules before colliding with another molecule is given by the mean free path,

(1) $$\lambda_{free}=\frac{k_{B}T}{\sqrt{2}\pi pd_{mol}^{2}},$$

where $k_{B}$ represents the Boltzmann constant, *T* is the absolute temperature, $d_{mol}$ is the kinetic diameter of the individual gas molecules and *p* denotes the pressure. The Knudsen number is defined as the ratio $\mathrm{Kn}\equiv \lambda_{free}/L_{c}$, with $L_{c}$ the characteristic length of the flow problem at hand. Situations with Kn>10 are said to be in the molecular flow regime, a situation commonly encountered in MEMS devices even at moderate vacuum levels because of the typically small characteristic lengths [10]. In the molecular flow regime, interactions between gas molecules can be neglected. When modeling the drag force on a body moving through a rarefied gas, we can therefore focus on individual collisions with the body’s surfaces.

#### 2.1.1. Damping of a Body in an Infinite Rarefied Gas

Assuming the velocities of the gas molecules follow a Maxwell–Boltzmann distribution, the force on a unit area (dA of a body moving through a rarefied gas can be calculated from integrating the momentum transferred by molecules colliding with this surface. A study by Martin et al. [11] suggests that these gas–wall collisions in microstructures in the free molecular flow regime are mostly diffuse (i.e., the outgoing velocity vector of a gas molecule is uncorrelated to its incoming velocity vector). Given that the velocity of the moving body, $\dot{y}$, is much smaller than the typical velocity of the gas molecules, the force on (dA can then be calculated to be [8]

(2) (dF⊥=−p(dA1+y˙⊥2m0πkBT1+π4,

where (dF⊥ denotes the force normal to the surface (dA, $m_{0}$ is the mass of a gas molecule and ${\dot{y}}_{⊥}$ is the component of body’s velocity normal to the surface (dA. The first term is just the force resulting from the average pressure on the unit area, but the second term is proportional to the body’s velocity and thus corresponds to a viscous drag force. Similarly, a drag force also results from the momentum transfer parallel to the unit area [8]

(3) (dF∥=−p(dAy˙∥m02πkBT

where ${\dot{y}}_{∥}$ denotes the component of the body’s velocity parallel to the unit area.

The net force on a moving body can be found by integrating the contributions in Equations (2) and (3) over its entire surface area. In the simple case in which all the surfaces are oriented either parallel or perpendicular to the direction of motion, the net force on the moving body can be found by adding the right contribution (perpendicular or parallel) for all its different surfaces. If we define $A_{x}$ as the total area of all surfaces that have their normal $\hat{n}$ aligned with the positive x-axis ($\hat{x}\;\cdot \;\hat{n}=1$) and define $A_{y}$ and $A_{z}$ similarly, the net force on a body moving along the y-axis is given by

(4) $$\begin{array}{c} \langle F_{net}\rangle =-\dot{y}\frac{8p}{\pi \bar{v}}\left(\frac{A_{z}+A_{x}}{2}+A_{y}\left(1+\frac{\pi }{4}\right)\right)\equiv -\dot{y}\gamma_{kin},\\ \mathrm{with}\;\bar{v}=\sqrt{\frac{8k_{B}T}{\pi m_{0}}}, \end{array}$$

where a factor 2 was added to account for all the matching surfaces that have their normals aligned with the negative axes. The contribution from the first term in Equation (2) cancels, because it is equal but oppositely oriented between the upwind and downwind surfaces. The resulting force is both proportional to and in antiphase with the body’s velocity and therefore corresponds to a viscous drag force. This drag effect is referred to as kinetic damping.

#### 2.1.2. Squeezed-Film Damping in a Rarefied Gas

In a typical MEMS device, moving structures are often surrounded by narrow gaps, especially if some form of capacitive readout is employed. The proximity of stationary structures close to a moving body contributes additional drag forces. In fact, this so-called squeezed-film damping often dominates the total gas damping. In the free molecular flow regime, the squeezed-film drag forces arise because of the time it takes an average molecule to diffuse out of the volume between a stationary and a moving surface. This makes it so that the body’s motion effectively modulates the local pressure inside these volumes. To quantify this effect, we can use a model proposed by Suijlen et al. [9] that considers the density of molecules in a volume between a stationary and a moving wall,

(5) $$n=\frac{N}{V}=\frac{N}{A(d_{0}-y)},$$

where *N* represents the total number of molecules present in a gap of total volume *V*, *y* denotes the wall displacement and $d_{0}$ and *A* are the equilibrium separation and the area of the gap walls, respectively. Because in general the gap is open, the total number of particles is not conserved, and a change in molecular density can generally be written as

(6) $$\Delta n=\frac{\partial n}{\partial N}\Delta N+\frac{\partial n}{\partial V}\Delta V=\frac{\Delta N}{Ad_{0}}+\frac{n_{0}}{d_{0}}y,$$

where $n_{0}$ is the equilibrium density of molecules inside the gap. If such a fluctuation in molecule density exists, particles will diffuse into or out of the gap. Defining τ to be the average time it takes for a molecule to diffuse out of the gap, the net number of particles leaving the gap per second is given by

(7) (d(dtΔN=−ΔNτ.

Using this result together with the ideal gas law $p=nk_{B}T$, we can take the time derivative of Equation (6) to write a differential equation for the pressure fluctuation inside the gap Δp

(8) (d(dtΔp=−Δpτ+p0d0(dy(dt,

with $p_{0}$ the equilibrium pressure. This equation is readily solved in the Laplace domain and gives an expression for the force on the moving wall as

(9) $$\begin{array}{cc} \Delta F(s)=-A\Delta P(s) & =-\frac{p_{0}A}{d_{0}}\frac{s\tau }{1+s\tau }Y\left(s\right)\\ \; & \approx -\frac{p_{0}A\tau }{d_{0}}\left(sY(s)\right)\;\mathrm{for}\;s\tau \ll 1, \end{array}$$

where *s* denotes the Laplace variable. This approximation is justified for typical MEMS mode frequencies, as we show below that, for a typical MEMS geometry, τ is of the order of a microsecond.

Equation (9) tells us that the squeezed-film damping force acting on the wall due to the modulated pressure inside the gap is both proportional to and in antiphase with the wall velocity sY(s), and therefore corresponds to a viscous damping effect with a coefficient

(10) $$\gamma_{sq}=\frac{p_{0}A\tau }{d_{0}}.$$

Typically, all areas in a MEMS sensor and their corresponding gap widths are known from the lithographic mask design. What is left to get an estimate for $\gamma_{sq}$ is to find an accurate estimate for τ.

### 2.2. Molflow+ Simulations

Estimating the diffusion time τ analytically for complex geometries is impractical, but, since in the molecular flow regime all interactions between different gas molecules can be neglected, the task is well suited for Monte-Carlo simulation. A Monte-Carlo simulator tool developed at CERN called *Molflow+* (v2.7.7) [12] was used to obtain the results presented here. This tool was originally developed for analyzing the vacuum system of particle accelerators [13], but it can also be used to simulate particle trajectories in arbitrary geometries [14].

#### 2.2.1. Geometry

**Figure 1:**
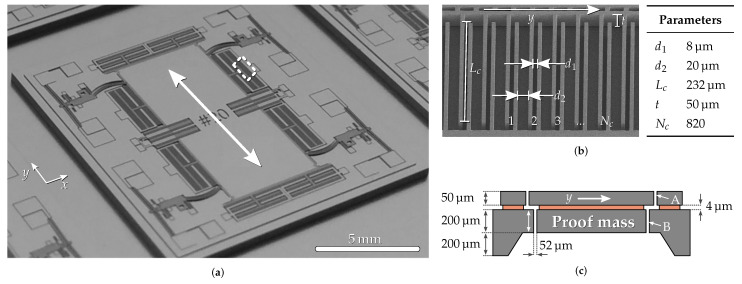
(**a**) Photograph of the MEMS accelerometer for which the gas damping is modeled. The central 12.7 mg proof mass can move along the y-direction as indicated by the arrow. (**b**) An electron microscope zoom-in of part of the MEMS sensing capacitors indicated by the boxed area in (**a**) along with the nominal etched dimensions. The edge of the proof mass is visible at the top of the image and can move along the indicated y-direction. (**c**) Schematic cross-section of the accelerometer. Significant squeezed-film damping occurs both in the capacitor structures in the top silicon layer (A) and in the bottom silicon layer (B).

Since in general the diffusion time τ will depend on the geometry of the gaps under consideration, a specific geometry needs to be chosen for simulation. The geometries that are considered here are part of the MEMS accelerometer shown in Figure 1a. The accelerometer is manufactured from a single silicon-on-insulator wafer and features a 12.7 mg proof mass suspended by a set of springs in each of its corners. The proof mass is surrounded by capacitive transducers for both position sensing and feedback actuation. All structures visible in Figure 1a are etched into the 50 μm thick top silicon layer, but most of the mass is actually contributed by silicon in the bottom layer suspended below it, as can be seen in Figure 1c. The main novelty of this accelerometer is its suspension spring system that can be used to significantly boost the sensitivity to acceleration. However, that feature is not relevant to the discussion about gas damping here, and the reader is referred elsewhere for more information [15].

With the proof mass moving along the y-direction, there are two regions in this device where significant squeezed-film damping occurs: (A) the narrow parallel plate capacitor gaps in the top silicon layer; and (B) the gap between the proof mass and the sensor’s frame in the bottom silicon layer. Monte-Carlo simulations were set up for both geometries. In the bottom silicon layer, there is only one 52 μm wide gap on either side of the proof mass, and, as shown in Figure 1b, the capacitors consist of many sets of parallel plates, representing a much larger total surface area. There are 410 of these sets of plates on either side of the proof mass for a total of 820 sets. They are positioned such that half of them are separated by a gap $d_{1}+y$ and the other half by $d_{1}-y$, so that a displacement *y* induces a differential capacitance change that can be measured to monitor the proof mass motion.

Next to the parallel plate capacitors that change their gap upon proof mass motion, the accelerometer shown in Figure 1a also contains capacitor fingers that change their overlap. Both the modulation of the volume between the static and moving parts and their surface areas along the direction of motion are significantly smaller than those of the parallel plate capacitor structures discussed above. Because of this, the expected squeezed-film damping from these comb finger structures is ≪1% of the total damping and was therefore neglected. These comb finger structures, however, do add kinetic damping that was accounted for by making sure to incorporate all contributing surfaces in the values for $A_{x}$, $A_{y}$ and $A_{z}$. Note that the walls of capacitive MEMS structures can generally exhibit significant scalloping as a result of the deep reactive ion etching process typically used to produce them. For the particular sensor in Figure 1a considered here, the impact of additional kinetic damping from the scallops on the total damping was estimated to be on the order of 1% and was not included. However, depending on the depth of the scallops and the total surface area of different geometries, it may be necessary to include the additional surface area created by these scallops in $A_{z}$ to get accurate results.

#### 2.2.2. Monte-Carlo Simulation Setup

**Figure 2:**
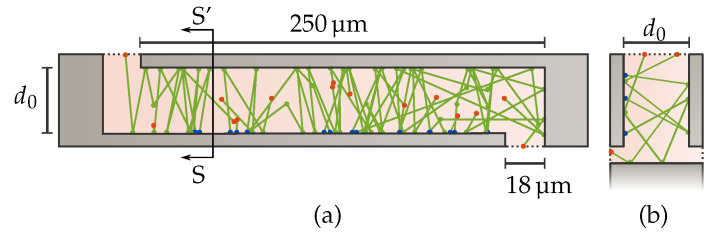
(**a**) Top view of typical 3D simulation result of a single set of capacitor plates ($d_{0}=30\;\mu m$). Particles spawn at one of the plates (blue dots) and are tracked through their reflections (green dots) until they leave the cavity (red dots). (**b**) Cross-section view SS’.

A schematic top view of the simulated sensing capacitor geometry is shown in Figure 2, where particles are emitted from one of the plates (blue dots). The molecules are traced along their random walk bouncing from all the different surfaces (green dots) until they leave the cavity (red dots). A particle is considered to have left the gap if it either moved out of the gap from the top or moved around or underneath the capacitor plate. In practice, this can be achieved by setting all these exit surfaces to absorb particles with 100% probability, terminating the particle’s flight once it hits one of them.

All surfaces were set to reflect diffusely with full thermal accommodation, meaning that, at every collision, the rebound velocity is determined from a Maxwell–Boltzmann distribution based on the surface temperature, and its direction is picked at random following a cosine distribution. In reality, a small fraction of all gas-surface interactions will be elastic. The tangential momentum accommodation coefficient (TMAC) that determines the fraction of all collisions that are diffuse depends on both the surface and the gas properties but is generally close to unity [16], justifying the assumption of fully diffuse interactions in the following simulations. A detailed study of the effects of the different accommodation coefficients on the total gas damping is beyond the scope of this study.

### 2.3. Quality Factor Measurements

To validate the Monte-Carlo assisted model, the resulting damping estimates are to be compared to quality factor measurements on the MEMS accelerometer in Figure 1. To avoid spurious excitation of the device’s main resonance mode during the measurements, the accelerometer was placed on a vibration isolation platform inside a large vacuum vessel [17]. Down to $10^{-4}$ mbar, the pressure inside the vessel was measured with capacitive membrane gauges (CMR261 and CMR365) to minimize the gas species dependence of the measurement. Below $10^{-4}$ mbar, a cold cathode gauge was used (IKR251).

**Figure 3:**
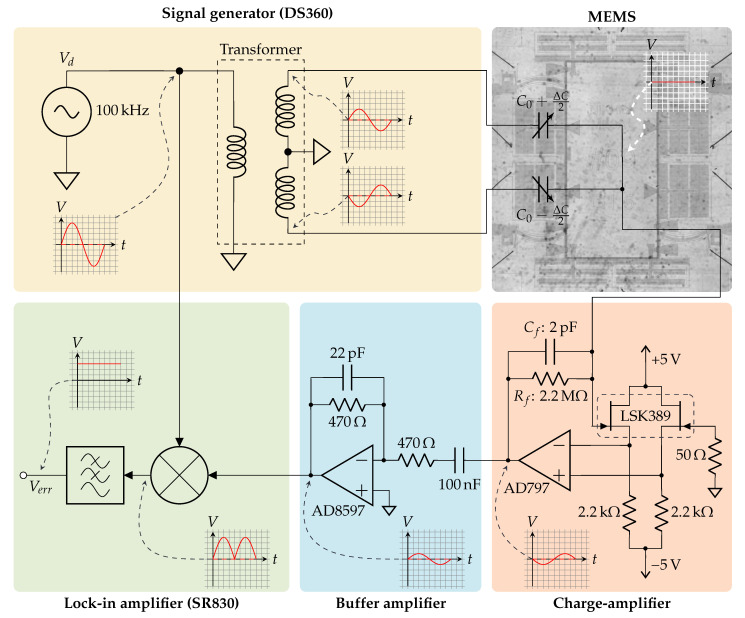
Schematic overview of the electronic system used to read the MEMS proof mass position. A transformer converts externally generated 100 kHz sine with amplitude $V_{d}$ to a balanced signal driving the MEMS capacitor bridge. The residual bridge current is integrated by a charge-amplifier, and its output voltage is buffered and sent to an external lock-in amplifier for synchronous demodulation. The circuit produces a low-frequency signal $V_{err}$ proportional to ΔC. The signals at different parts of the circuit are indicated.

To measure the motion of the accelerometer’s proof mass, the sensor was interfaced to the capacitive half-bridge readout, as shown in Figure 3. This readout system drives the capacitor bridge in the accelerometer with a differential signal at 100 kHz and collects any mismatch current through a charge-amplifier that is electrically connected to the proof mass through its suspension springs. Synchronous demodulation with a lock-in amplifier results in a signal $V_{err}$ that is proportional to the proof mass displacement from equilibrium, as long as the displacement is kept sufficiently small to avoid significant non-linearities being introduced by the capacitor bridge.

Next to reading its position, a force can be exerted on the proof mass by applying a voltage to a separate set of capacitive transducers. Using this setup, the device’s quality factor can be determined by electronically exciting the main resonance mode, turning off the excitation source and recording the subsequent exponential decay.

## 3. Results

### 3.1. Simulation Results

**Figure 4:**
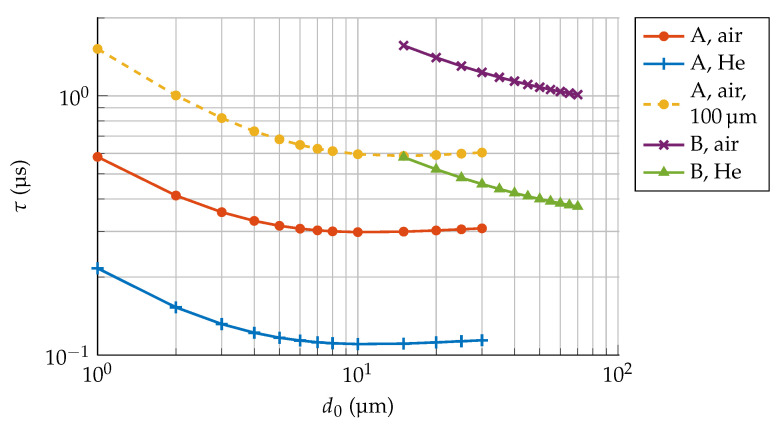
Molflow+ simulation results for the diffusion time τ as a function of plate separation $d_{0}$ for air and helium particles at 300 K, escaping from regions A and B as indicated in Figure 1c. The dashed curve corresponds to particles escaping from region A, but with a 100 μm thick silicon layer.

A histogram of the flight time of all particles in the Monte-Carlo simulation is recorded, from which the average diffusion time τ can readily be calculated. Simulations were performed for a range of plate separations $d_{0}$, for both the capacitor structures and the gap at the faces of the proof mass, indicated as A and B in Figure 1c, respectively. To study the effect of different gas species, all geometries were considered both in air ($m_{0}=28.97\;u$) and in helium ($m_{0}=4.00\;u$) at 300 K, and the results are summarized in Figure 4. Each data point was obtained from a Monte-Carlo simulation with $O(10^{6}$–$10^{7})$ simulated particles.

Since the expected path a gas molecule takes does not depend on the gas species, the average flight distance for all particles is the same. The diffusion times for air and helium are therefore expected to differ only due to their difference in average particle velocity $\bar{v}$ as defined in Equation (4). The ratio of these average velocities is simply given by $\sqrt{m_{air}/m_{He}}\approx 2.69$, and indeed the results shown in Figure 4 for helium and air differ by this factor to well within 1%.

### 3.2. Experimental Verification

#### 3.2.1. Pressure Dependence of the Quality Factor

**Figure 5:**
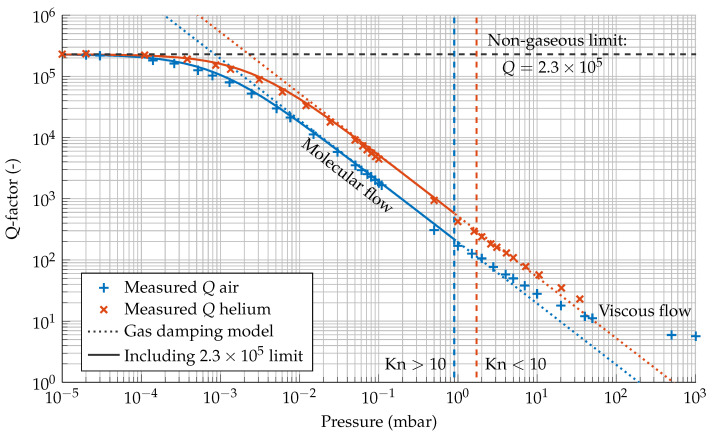
*Q* factor measurements for a MEMS accelerometer with a natural frequency of 183.3Hz in air and helium atmospheres at different pressures. The dotted curves represent the expected *Q* from the total modeled gas damping $\gamma_{tot}$ as a function of pressure. The model is valid for Kn>10. The pressure was measured with two capacitive gauges, a CMR261 ($10^{-1}\mathrm{mbar}$ to $10^{3}\mathrm{mbar}$) and a CMR365 ($10^{-4}\mathrm{mbar}$ to $10^{-1}\mathrm{mbar}$), and a cold cathode gauge, IKR251 ($<10^{-4}\mathrm{mbar}$).

All relevant surface areas of the MEMS accelerometer for calculating the damping contributions are listed in Table 1. Using these parameters together with the estimates for τ obtained through Monte-Carlo simulation, the total expected gas damping as a function of pressure can be calculated through Equations (4) and (10). The relative contributions of both kinetic damping and squeezed-film damping from distinct regions in the accelerometer are listed in Table 2, as well as the corresponding damping coefficient at a reference pressure of 0.1 mbar. Squeezed-film damping from region B is relatively small, since the gap is both wider and the contributing surface area is significantly smaller as compared to region A. Note that the kinetic damping constitutes 28% of the total gas damping. This contribution is often assumed to be negligible with respect to squeezed-film damping, but, especially for bodies with a relatively large surface area such as the proof mass considered here, this is not always justified. For comparison with experimental results, the damping contributions are converted to quality factors as

(11) $$Q=\frac{2\pi mf_{0}}{\gamma_{tot}},$$

where $f_{0}$ denotes the accelerometer’s natural frequency and *m* is its proof mass. Figure 5 shows the quality factors expected from the model as a dotted curve along with quality factor measurements as a function of pressure. The solid curve represents the model prediction when the effect of the non-gaseous limit of $Q=2.3\times 10^{5}$ is included. The experimental results were obtained from measuring free exponential decays in both air and helium atmospheres at different pressures.

At atmospheric pressure, we have Kn≪10 and the molecular flow assumption used to construct the viscous damping model is invalid. Lowering the pressure to below about 1 mbar, we have Kn>10, and the measured *Q* values converge to the ones predicted from gas damping to within about 15%. Note that the characteristic length $L_{c}$ differs between different regions in the MEMS device, so they all have their own slightly different Kn. The Kn=10 limits in Figure 5 are calculated for $L_{c}=8\;\mu m$ as that gap contributes the largest part of the damping. Below a pressure of $10^{-3}\mathrm{mbar}$ , gas damping is no longer dominant and *Q* converges to 2.3 × 10^5^, a value set by a combination of internal friction in the suspension system and other unmodeled losses.

#### 3.2.2. Dependence on Plate Separation

Next to a dependence on pressure, the squeezed-film damping as modeled in Equation (9) shows a direct dependence on the equilibrium wall separation $d_{0}$. Since the proof mass in the MEMS accelerometer has a movement range of ±5 μm, by virtue of its relatively low natural frequency, $d_{0}$ can be modulated by this amount by simply tilting the accelerometer with respect to gravity by an angle θ. The static proof mass displacement *y* can then directly be obtained from $y=g\sin\left(\theta \right)/{\left(2\pi f_{0}\right)}^{2}$, with θ=0 indicating the orientation in which the accelerometer’s sensitive *y*-axis is oriented orthogonal to local gravity *g*.

**Figure 6:**
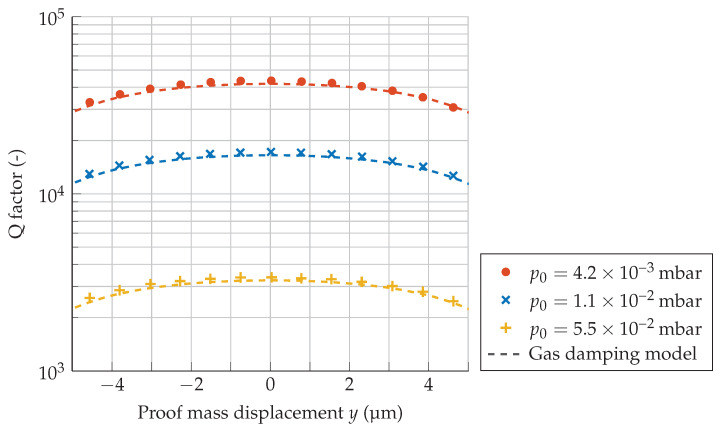
*Q* factors in air as a function of proof mass displacement for a MEMS accelerometer with $f_{0}=164.0\mathrm{Hz}$.

Figure 6 shows three series of quality factor measurements as a function of proof mass displacement for different vacuum levels, for which Figure 5 tells us that the damping is dominated by gaseous effects in the free molecular flow regime. Additionally, the expected quality factor values from the total modeled gas damping, $\gamma_{kin}$ and $\gamma_{sq}$, are plotted as a function of the proof mass displacement *y*. The model curve for *Q* was obtained by replacing $d_{0}$ with $d_{0}\pm y$ for the relevant gaps. The geometry dependence of τ from the simulations in Figure 4 is taken into account when plotting the model, and the resulting curve agrees with the measured data to within 5% over the entire measured range.

## 4. Discussion

The simple analytical model that leads to the estimate for squeezed-film damping in Equation (10) is quite powerful. It reduces all the geometry dependencies that cannot be easily included analytically to a single parameter τ, which lends itself to be accurately estimated by Monte-Carlo simulation. This approach has some benefits over the more commonly adapted Monte-Carlo method that monitors the momentum transferred during individual wall-molecule collisions [7]. Firstly, for estimating τ, there is no need to consider the motion of the geometry under consideration, whereas getting an estimate for the damping through tracking the momentum transferred in individual collisions requires doing so through multiple oscillation periods. This comes at a significant additional computational expense. Secondly, since there is no need to monitor the momentum transfer to the wall that is typically moving slow in comparison to the gas molecules, this method does not suffer from the statistical noise that is typically encountered in Monte-Carlo methods that simulate gas damping [3]. Moreover, estimating τ can be done with e.g., Molflow+, which is openly available, thus requiring no custom algorithm development. The match between the measured quality factors and the modeled damping obtained in this way is better than 15% for all measured parameter combinations within the range where the model is valid. This level of accuracy is comparable to that of other simplified damping models, for example that of Fedeli et al. [18] that had its accuracy benchmarked against data from an extensive experimental campaign [19].

The estimates for the diffusion time τ, as shown in Figure 4, were determined under the assumption that the walls of the simulated geometry are smooth. In reality, however, the etched surfaces of a capacitive transducers will exhibit some amount of roughness and scalloping. Several analyses [20,21] suggest that these surface properties can slightly increase τ. When the scalloping profile that results from the fabrication process is known, it can be added to the simulation geometry to improve the accuracy of the estimates for τ at the cost of added model complexity.

While Figure 4 shows simulation results for both air and helium atmospheres, it is really only necessary to consider one of them as the results only differ by their mass ratio as $\sqrt{m_{air}/m_{He}}$. This means that τ can be estimated through Monte-Carlo simulations for a single arbitrary gas species, and the results can be scaled to accurately predict the gas damping of any mix of different gases, as long as the composition is known. This can be useful for MEMS devices that have been encapsulated in a vacuum with a getter material, as the atmosphere inside those cavities usually does not resemble air and has a high noble gas content against which the getter material is not effective [22].

In an accelerometer such as the one considered here, the Brownian noise floor is determined by the total gas damping, of which the largest part consists of squeezed-film damping in the capacitor structures. The geometry of these capacitors is usually dictated by electrical requirements, but it is insightful to look at how it affects the Brownian noise level as well. The results in Figure 6 show that Equation (10) accurately describes the dependence of the squeezed-film damping on the wall separation as $1/d_{0}$. This happens to be the same scaling as for the capacitance of parallel plates $C=\epsilon_{0}A/d_{0}$. Normalizing by *C*, the squeezed-film damping per unit of capacitance is

(12) $$\frac{\gamma_{sq}}{C}=\frac{p_{0}A\tau }{d_{0}}\frac{d_{0}}{\epsilon_{0}A}=\frac{p_{0}}{\epsilon_{0}}\tau .$$

This quantity depends on the capacitor geometry only through τ, meaning that, for a given capacitance, the capacitor geometry can be optimized for electrical performance with minimal impact on Brownian noise as long as τ remains constant. The curves in Figure 4 show that, in the considered capacitor geometry, τ changes by less than 10% for values of $d_{0}$ ranging from 4 μm–30 μm. Using any value for $d_{0}$ in this range will provide virtually the same Brownian noise level for a given sensing capacitance.

Although there are benefits to using a thicker silicon device layer, the result in Equation (12) also exposes one of the disadvantages of doing so. The simulations in Figure 4 show that the values for τ are 2.0–2.6 times larger for a 100 μm thick device layer than for a 50 μm thick one, meaning that, for the same sensing capacitance, the former will have roughly twice as much squeezed-film damping.

## 5. Conclusions

An analytical model for gas damping in the free molecular flow regime of both kinetic and squeezed-film origin was used to predict the quality factor of a MEMS accelerometer in both air and helium atmospheres. This prediction matches the experimental results to within 15% in the pressure range where the model is valid (i.e., Kn>10). The model for squeezed-film damping conveniently reduces the unknown geometry dependence to a single parameter τ that can accurately be estimated through Monte-Carlo simulation. As long as the gas composition is known, a single simulated result for τ can be scaled to predict the damping from arbitrary gas mixtures.

The dependence of the squeezed-film damping on the wall separation as predicted by the analytical model was verified experimentally. The model matches the experimental results to within 5% over the entire range of wall separations that was tested at three different pressures.

The gas damping that ultimately leads to Brownian noise in the MEMS accelerometer considered here is dominated by squeezed-film damping in the capacitor structures. For a given capacitor plate height, this squeezed-film damping is inversely proportional to the plate separation $d_{0}$ over a wide range, which is the same scaling as for the capacitance itself. This implies that, for a given transducer capacitance, its geometry can be optimized for the best electrical performance without ramifications for the sensor’s Brownian noise performance.

## Figures and Tables

**Table 1:** Relevant areas in the accelerometer in Figure 1a.

	Value	Description
$A_{x}$	$6.42\times 10^{-6}$ m ${}^{2}$	Total area with normal along $+\hat{x}$
$A_{y}$	$1.26\times 10^{-5}$ m ${}^{2}$	Total area with normal along $+\hat{y}$
$A_{z}$	$2.50\times 10^{-5}$ m ${}^{2}$	Total area with normal along $+\hat{z}$
$A_{8}$	$9.51\times 10^{-6}$ m ${}^{2}$	Total area facing 8 μm gap (A)
$A_{20}$	$9.51\times 10^{-6}$ m ${}^{2}$	Total area facing 20 μm gap (A)
$A_{B}$	$1.23\times 10^{-6}$ m ${}^{2}$	Total area facing 52 μm gap (B)

**Table 2:** Damping contributions in air ( 300 K, 0.1 mbar).

	Value		Description
$\gamma_{kin}$	$2.08\times 10^{-6}$kg/s	(28%)	Kinetic damping
$\gamma_{sq,8}$	$3.57\times 10^{-6}$kg/s	(49%)	Squeezed-film A, 8 μm gap
$\gamma_{sq,20}$	$1.44\times 10^{-6}$kg/s	(20%)	Squeezed-film A, 20 μm gap
$\gamma_{sq,B}$	$0.25\times 10^{-6}$kg/s	(3%)	Squeezed-film B
$\gamma_{tot}$	$7.34\times 10^{-6}$kg/s	(100%)	Total gas damping

## Data Availability

Not applicable.
